# Analysis of Heart Rate, Perception of Physical Effort and Performance of Individuals with Down Syndrome Submitted to a Protocol of Virtual Games for Home-Based Telerehabilitation

**DOI:** 10.3390/healthcare11131894

**Published:** 2023-06-30

**Authors:** Renata Martins Rosa, Maria Helena Santos Tezza, Elisa de Jesus Valenzuela, Eduardo Dati Dias, Íbis Ariana Peña de Moraes, Luciano Vieira de Araujo, Alessandro Hervaldo Nicolai Ré, Talita Dias da Silva, Carlos Bandeira de Mello Monteiro

**Affiliations:** 1 Postgraduate Program in Rehabilitation Sciences, Faculty of Medicine, University of São Paulo (FMUSP), São Paulo 01246-903, SP, Brazil; 2 Postgraduate Program in Physical Activity Sciences, School of Arts, Science and Humanities of University of São Paulo (EACH-USP), São Paulo 03828-000, SP, Brazil

**Keywords:** Down syndrome, virtual reality, rehabilitation

## Abstract

Down syndrome (DS) is a genetic condition associated with impairments in several body systems, which may negatively influence the habit of practicing physical activities (PAs), increasing sedentary habits and the risk of comorbidities. Additionally, difficulty in accessing services, financial limitations and lack of interest may interfere with the practice of PAs. Considering the necessity of developing effective treatment alternatives, to increase the possibility of access and the interest of participants, we conducted a study using telerehabilitation with a virtual task to promote PA and analyze the motor performance of DS individuals. Our protocol consisted of 11 sessions of the virtual game called MoveHero. A total of 34 individuals with DS and 34 individuals with typical development participated in the study. Heart rate (HR) and rating of perceived effort (RPE) were collected at rest and during the game. Our results show that virtual reality presents a great possibility to promote PA and a way out of a sedentary lifestyle for DS individuals, considering the enhancement in HR and RPE found during the protocol for both groups. Moreover, our results show positive outcomes regarding motor performance, with significant improvement in the task with practice, demonstrating that individuals with DS are able to improve their motor proficiency with adequate stimuli in the virtual environment.

## 1. Introduction

Down syndrome (DS) is one of the most common chromosomal abnormalities of childhood (chromosome 21 trisomy), causing possible impairments in mental, sensory, and cardiovascular systems, as well as deficiencies in neuromusculoskeletal and movement-related functions [1,2]. Neuromusculoskeletal dysfunctions, such as hypotonia, joint hypermobility, and ligament laxity, may negatively influence the habit of practicing physical activity (PA), contributing to a sedentary lifestyle in this population [1,2]. Moreover, individuals with intellectual disabilities are more sedentary than their peers without disabilities [3,4], and decreased PA in the DS population has been found to result in functional activity impairments, contributing to several comorbidities both in childhood and in adult life [5].

The reasons for the inactivity in DS are multifactorial, including physiological causes (such as neuromusculoskeletal or cardiovascular) and behavioral characteristics [4]. According to Grieco et al. [6], the presence of intellectual disability, difficulties in staying engaged with a task, a tendency to consistently respond in the same manner to certain situations, and a slower reaction time are some of the phenotypic characteristics of DS that may contribute to impaired functioning during their lifespan [6]. These characteristics can also contribute to lower levels of engagement in PA, which could improve functional ability and reduce general health risks for this population [5]. 

Fox et al. [5] reported that improvement in cardiorespiratory fitness, development of a strong musculoskeletal system, and reduced risks of developing health conditions, such as heart disease, cancer, diabetes, high blood pressure, and obesity, are some of the benefits of regular PA practice. The World Health Organization advocates that children and adolescents should practice at least 60 min of moderate to vigorous PA during the week, preferably aerobic activities. The same recommendation is given to children and adolescents with disabilities [7]. 

In addition to the known difficulty of promoting PA for individuals with DS, in 2020, the world experienced the need for quarantine and received several recommendations from global health institutions for social distancing [8,9]. Despite the distancing recommendations, it was necessary to make every effort to maintain adequate levels of PA for individuals with therapeutic needs [10], including individuals with DS. 

Thus, the use of new technologies, such as home-based telerehabilitation (HBTR) [11], can provide continuous interventions and be considered in future PA interventions. HBTR is an effective strategy that uses technology to promote remote rehabilitation, allowing patients to have a more fun and engaging experience [5,12].

In the context of HBTR, the development of new interventions using technology, such as virtual reality (VR) exergames, is an interesting and effective approach [13] as an adjuvant treatment to continue rehabilitation or to maintain its benefits for various neurological difficulties, including DS [14]. VR exergames are characterized as active games, with whole-body movements, requiring energy expenditure similar to moderate-intensity PA [15,16]. In addition to the benefits of providing physical activity with the use of virtual tasks, there is the possibility of improving motor performance during practice of the activity. According to Monteiro et al. [14], the observation of how motor ability may be improved with the practice of exergames could also have important practical implications for rehabilitation programs for those affected by DS, providing the possibility of intervention programs based on evidence of good outcomes using this modality [14]. 

Considering the above, we organized a study with individuals with DS and typical development (TD) individuals to investigate the effects of an HBTR protocol (based on VR exergames) as an alternative to PA practice, analyzing the increase in heart rate (HR) and rate of perceived effort during practice (RPE), as well as the possibility of improving the motor abilities of the players (verified by the game performance). 

We hypothesized that both the participants with TD and with DS would present an increase in HR, RPE, and performance improvement during practice; however, the DS individuals would present higher values of HR and RPE because of sedentarism and hypotony, as well as lower performance during the game due to motor and cognitive difficulties, such as the pattern of slow movements that characterizes DS.

## 2. Materials and Methods

### 2.1. Study Design

We registered this trial at ClinicalTrials.gov (RBR-5rsrbk6). This paper was reported in accordance with the Standard Protocol Items: Recommendations for Interventional Trials (SPIRIT). We divided our study into two phases, presented as follows:

(1) The first phase consisted of DS and TD individuals (paired by sex and age) performing one day of the game task for 20 min (with three matches of the game), and comparisons between the groups in terms of HR, RPE, and motor performance were made during the practice.
(2) The second phase, only for the DS individuals, consisted of a 10-day practice protocol in addition to the first day, scheduled two times a week according to participant availability (thus, the protocol for DS individuals consisted of 11 days): participants played the game for approximately 20 min each day (with three matches of the game), and their HR and RPE during the practice were registered. After the 10th session of the protocol, the participants were allowed a 15-day break before another day of practice was scheduled with the intention of analyzing whether participants maintained their performance.

### 2.2. Participants

Participants were recruited in Brazil through social media (with the propagation of the main project information—such as the aim of the study; population and ages of interest) and through networks (indicated by other health professionals working in the pediatric field). Family members or DS individuals with interest in participating got in touch with the researcher through a phone number that was disclosed along with the project information or through the researcher’s social media profile (also disclosed with the project information). In the case of availability to participate and acceptance by family members and participants, the consent form (for participants over 18 years of age or the legal guardians of the younger participants) and/or the assent form (for participants younger than 18 years of age) was explained and sent for their agreement. The criteria for inclusion were a previous diagnosis of DS, completed terms of agreement to participate in the study signed by the participants (assent form) and their legal guardians (consent form), and age ≥ 10 years, with the ability to understand the proposed task. The exclusion criteria were disorders in cognitive function that would prevent comprehension of the task instructions and technical issues with computers or the Internet.

### 2.3. Assessment Instruments

All assessments were conducted through online meetings with the participants and their family members. The Pediatric Evaluation of Disability Inventory (PEDI) questionnaire was used to document the functional profile of the DS individuals, which was adapted to the Brazilian population [17]. The questionnaire provides information about three functional areas: self-care, mobility, and social function, as well as data on how independent the patient is or whether they require assistance from caregivers or modifications to the environment [17].

For both the DS and TD participants, the International Physical Activity Questionnaire (IPAQ), a reliable instrument to measure self-reported PA, was used to document and characterize the level of PA practiced in their routine. Based on the participant’s responses, the questionnaire allocates them one of four classifications: active, irregularly active, very active, or sedentary, according to the frequency and intensity of PA in their routine [18]. Participants were also questioned concerning how many hours per day they generally spend using specific electronic devices. 

**Figure 1:**
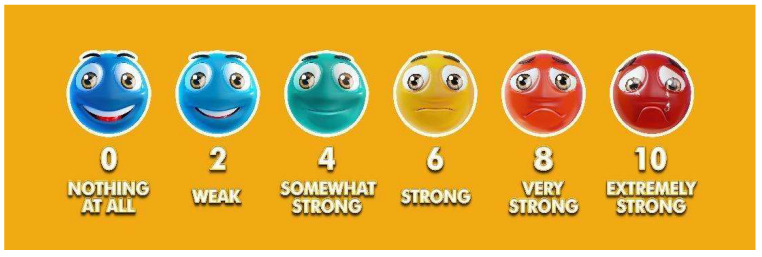
The Visual Rating of Perceived Exertion (RPE) based on Nashimoto et al. [19].

To assess the perception of physical effort before and during the intervention, we used the Rating of Perceived Exertion (RPE). A visual scale was presented to the participants (Figure 1), and they were asked to quantify the sensation of effort at that moment [19]. The visual component facilitates understanding of the question regarding their level of tiredness for children and individuals with cognitive disabilities and refers to the commonly used Borg category-ratio 10-point scale, with the addition of facial expressions (i.e., smileys) for the RPE scores of 0, 2, 4, 6, 8, and 10 [20]. The Physical Activity Guidelines for Americans correlates the RPE classification with the intensity of PA, with moderate-intensity activities scoring 5 or 6 and vigorous-intensity activities 7 or 8 [21]. 

Heart rate (HR) was measured with a pulse oximeter or a smart watch if available in the participant’s home, or, in the absence of both, the researcher explained how to use the Instant Heart Rate^®^ application, available for Android and IOS for free, which captures the HR measurement when positioning the participant’s finger on the cellphone camera [22]. The Instant Heart Rate^®^ is a valid and reliable tool to assess pulse rate and uses the phone’s camera lens and flash to measure the pulse in the index finger of the individual [22]. 

Motor performance was analyzed considering the game error variables (absolute error and variable error), provided by the game software, which report the accuracy and precision of moments. The absolute error (AE) is the representation of the movement accuracy, and the variable error (VE) is the representation of the precision of movements (see Monteiro et al., 2017 [14]). 

### 2.4. Intervention Protocol: MoveHero 

**Figure 2:**
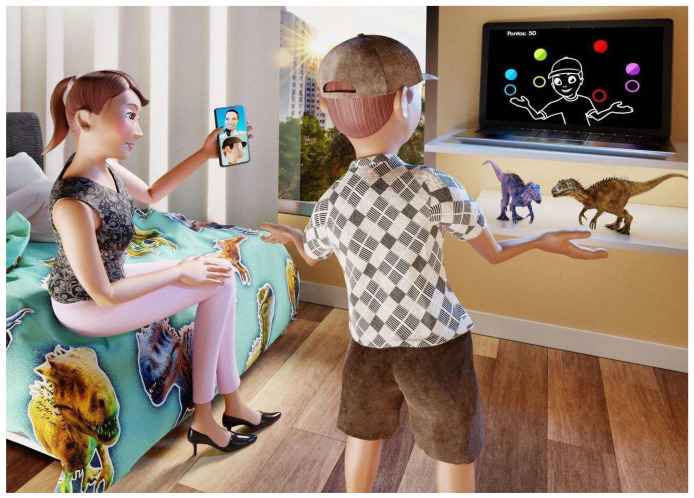
Illustrative picture of a participant playing the MoveHero game.

The MoveHero software is considered a timing coincident task game (Figure 2), used on a computer or tablet, with spheres that fall down the device’s screen in four columns (two columns on the right and two on the left). Each mobile sphere has a fixed target (fixed spheres at the end of the columns), and participants are required to move their left upper limb or right upper limb when the mobile spheres reach the fixed targets, directing the corresponding upper limbs to each column. We used this software for 20 min of practice, because it is considered a laboratory-specific motor task and has been used in several studies with motor learning protocols and showed positive outcomes in cerebral palsy [23], spinal cord injury [24], and amyotrophic lateral sclerosis (ALS) [25].

The participants were positioned in front of the computer, between the two central spheres, making sure that their shoulders or head did not touch these spheres virtually. When the game starts, the webcam captures their movements, and a representation of the player appears on the computer screen as an avatar, who performs the virtual contact. The task is to intercept all the falling spheres using upper limb movements at the exact moment the spheres reach their specific fixed target at the bottom of the screen. The game provides feedback on hits and misses by changing the color of the spheres (green for a hit and red for a miss).

### 2.5. Procedures

After the first contact between the researcher and family/participant through social media, the project was explained in full at an online meeting, and those who met the inclusion criteria were invited to sign the consent and/or assent form. The next step was to schedule the first day of the protocol (also through an online meeting), which consisted of the execution of the assessments (as described in Section 2.3) and then to start the practice. To participate, it was only necessary to have a computer with webcam and access to the internet. The participants were allocated in their own houses, and the researcher showed the participants how to enter the website of the game and simulated a match so that the participants understood the task, showing them how to play and the necessary position adjustments: player’s head and shoulders centralized in front of the camera (between the central spheres on the screen), and arms so that, when opened, they touched the left- and right-side columns. The researcher instructed the family members and/or participants on measuring HR: use of the pulse oximeter or smart watch, if available, and in the absence of both, how to download and use the Instant Heart Rate^®^ application.

**Figure 3:**
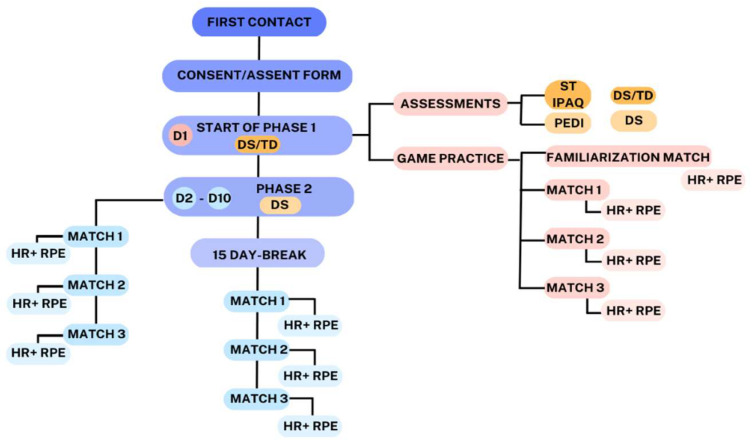
Flowchart of the procedures of the study. D1—Day 1 of the protocol; D2–D10—from day 2 to day 10 of the protocol; DS—Down syndrome; TD—typical development; ST—screen time; IPAQ—International Activity Questionnaire; PEDI—Pediatric Evaluation of Disability Inventory; HR—heart rate; RPE—Rating of Perceived Effort.

After this process, a familiarization match began (in which the participant played the game to verify if they really understood the game task). The game task was performed with the virtual game called MoveHero (detailed in Section 2.4), and while participants were playing the game, the researcher observed online and made necessary adjustments, also registering their HR and RPE measurements between matches (details on the instruments used are provided in Section 2.3). We conducted three matches per day, lasting approximately five minutes each. Between matches, the researcher used the RPE to verify their perceived level of effort and registered their HR. The measures of RPE and HR were registered at rest (pre-activity) and after each game match (every 5 min, until three matches were completed). Figure 3 below shows a flowchart of the procedures.

### 2.6. Statistical Analysis

Descriptive statistics were performed. Categorical data are reported as the absolute and relative frequencies, while continuous data are reported as the mean and standard error (SE) using graphs and in Tables S1 and S2 (Supplementary Materials). The normality of the data was assessed using a histogram with an analysis of the normality curve. Linear mixed models (LMMs) were used to compare the HR, RPE, AE, and VE variables between groups (TD and DS) for four repeated measurements (Rest or Match 0, Match 1, Match 2, and Match 3). We also used LMMs to determine the mean changes in the HR, RPE, AE, and VE variables in the DS group (within group) through the days of the second phase of the protocol. The least significance difference (LSD) was used as a post hoc test to determine the differences found in the LMM effects and interactions.

## 3. Results

A total of 68 individuals participated in the study, 34 with DS (mean age: 18.9 ± 6.1) and 34 with TD (mean age: 19.0 ± 1.6), paired by sex and age. The participants were of both sexes (18 males, 16 females). All participants in the TD group and 32 in the DS group were from different states of Brazil (participants lived in different geographic regions within them). All participants in both groups had previous contact with technology, and 86.7% reported a screen time of over 10 h per week. None of the participants had previous contact with VR in rehabilitation programs. Table 1 presents the characteristics of the included participants. The results of the protocol are presented in two phases: first, comparing the performance (AE and VE), HR, and RPE between the two groups; second, presenting the DS results over the 11 days of the protocol. 

### 3.1. Heart Rate (HR)

**Figure 4:**
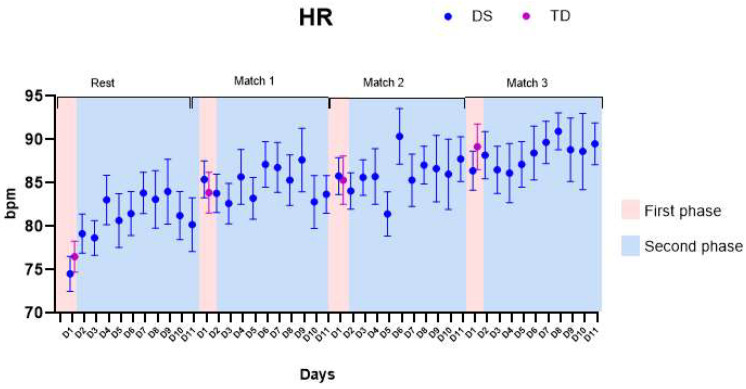
Heart rate (HR). DS—Down syndrome; TD—typical development; D1—day 1.

Figure 4 presents then HR, considering both the first phase (pink color on the graph) and the second phase of the protocol (blue color on the graph).

(1) First phase: No significant differences were found comparing the HR of both groups (the DS group presented average increases of 13.7 ± 2.4 beats per minute (bpm), and the TD group presented average increases of 12.7 ± 3.2 bpm). We found main effects for matches within-groups: both DS and TD enhanced their HR values comparing rest with Match 1 and Rest with Match 3 (*p*-values and descriptive statistics are provided in Table S1).
(2) Second phase: We found main effects for Matches (*p*-values and descriptive statistics are provided in Table S2). No significant differences were found for HR comparing the days of the protocol.

### 3.2. Borg Rating of Perceived Exertion (RPE)

**Figure 5:**
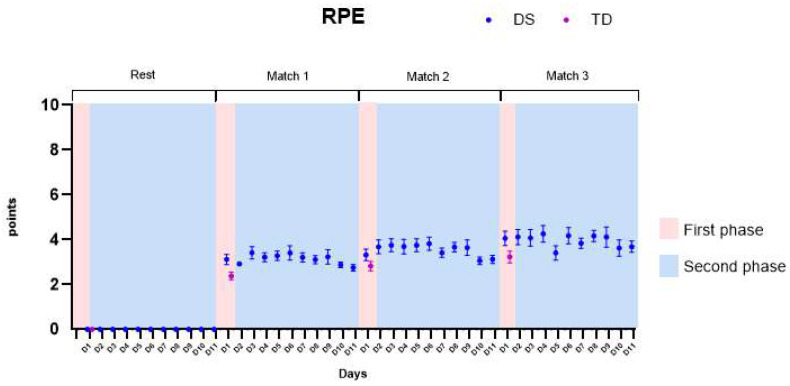
Borg Rating of Perceived Effort (RPE). DS—Down syndrome; TD—typical development; D1—day 1.

Figure 5 presents the RPE, considering both the first phase (pink color on the graph) and the second phase of the protocol (blue color on the graph).

(1) First phase: We found main effects for groups and in the interaction groups by matches. The DS group presented significantly higher values of RPE when compared to the TD group, presenting higher values of RPE in the first (M1—mean difference of 0.8 ± 0.2), second (M2—mean difference of 0.4 ± 0.2), and third matches (M3—mean difference of 0.7 ± 0.2). All *p*-values and descriptive statistics are provided in Table S1.
(2) Second phase: We found significant increases in RPE in the DS individuals when comparing matches. The differences were also found when comparing the first day (D1) and the last day of the protocol (D11), enhancing the RPE. All *p*-values and descriptive statistics are provided in Table S2.

### 3.3. Absolute Error (AE)

**Figure 6:**
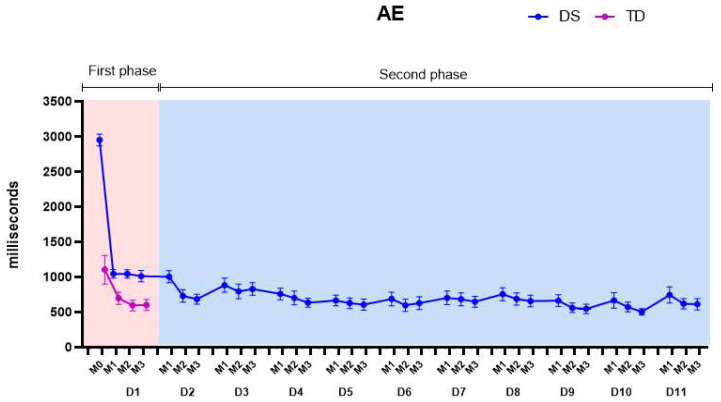
Absolute errors (AEs). DS—Down syndrome; TD—typical development; M1—Match 1; M2—Match 2; M3—Match 3; D1—day 1.

Figure 6 presents the AE, considering both the first phase (pink color on the graph) and the second phase of the protocol (blue color on the graph).

(1) First phase: We found main effects for groups, matches, and for the groups by matches interaction. Comparisons between groups showed that the DS group started the practice with higher mean values of AE than the TD group, with a mean difference between groups of 763.2 (±54.9) ms (*p*-values and descriptive statistics are provided in Table S1).
(2) Second phase: Significant differences were found comparing the days and matches of the protocol. On the fourth day of practice, the AEs of the DS group reached the values of the TD group and maintained the decrease in errors, presenting lower values than the TD group from the fifth day on (639.0 ± 53.2 ms). A significant decrease in the AEs was found comparing D1 with D10 and D1 with D11. All *p*-values and descriptive statistics are provided in Table S2.

### 3.4. Variable Error (VE)

**Figure 7:**
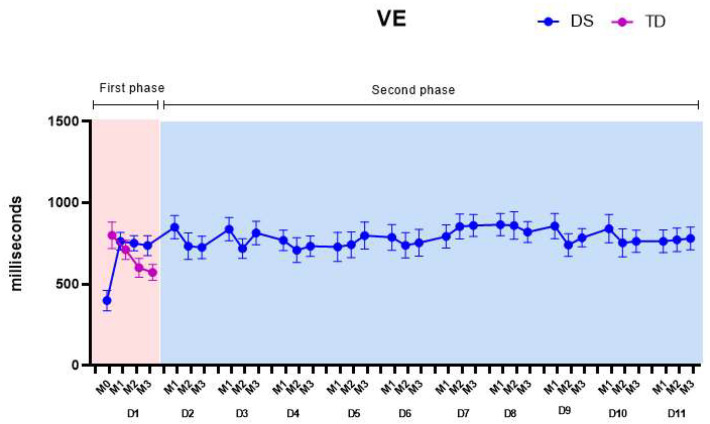
Variable errors (VEs). DS—Down syndrome; TD—typical development; M1—Match 1; M2—Match 2; M3—Match 3; D1—day 1.

Figure 7 presents the VE, considering both the first phase (pink color on the graph) and the second phase of the protocol (blue color on the graph).

(1) First phase: We found main effects for matches and in the groups by matches interaction. Comparing the familiarization match (M0) between the DS and TD groups, the DS group showed significantly lower values (363.9 ± 57.1) of VE than the TD group (771.4 ± 69.1), which is the opposite of what occurred for the AE. All *p*-values and descriptive statistics are provided in Table S1.
(2) Second phase: Over the days, the mean VE values of the DS group increased, varying between 770.4 ± 44.6 ms and 849.3 ± 45.3 ms but not reaching the mean values of the TD group (664.6 ± 30.4 ms). These results are further discussed below. All *p*-values and descriptive statistics are provided in Table S2.

## 4. Discussion

In the current study, we investigated the influence of a nonimmersive VR telerehabilitation intervention in individuals with DS with the aim of promoting an alternative to PA practice (analyzing whether playing the virtual task would enhance HR and RPE) and to investigate if DS individuals would reach the motor performance of TD individuals in the virtual task. 

We hypothesized that both the participants with TD and with DS would present an increase in heart rate (HR), rate of perceived effort (RPE), and performance improvement during practice, but the DS individuals would present higher values of HR and RPE due to sedentarism and hypotony and lower performance during the game because of motor and cognitive difficulties, such as the pattern of slow movements that characterizes DS.

Confirming our hypothesis, we identified that both groups (DS and TD) presented an increase in heart rate (HR) and in the Rating of Perceived Effort (RPE) with game practice and that DS individuals presented higher values of HR and RPE compared with TD individuals. Additionally, considering performance during the game, we observed that the DS group started the protocol with worse performance than the TD individuals, and improved over the days of practice, reaching similar results as the TD group in the game variables analyzed. We discuss these findings below.

### 4.1. First Phase: Heart Rate (HR) and Rating of Perceived Effort (RPE) with Game Practice

Our results show that both groups (TD and DS) presented incremental values of HR while playing the game. However, no significant differences were found between the increase in HR when comparing the groups, and both presented similar patterns of HR increase. On the other hand, the DS group presented higher RPE values while playing compared with the TD group. Practice enhanced the DS individual’s perception of effort, and although both groups reported some level of tiredness when practicing the VR task during the matches, greater intensity was reported by the DS group. 

Although the reported perception of effort increased in both groups, the TD group did not reach sufficient intensity to consider the virtual game as moderate-intensity PA practice, taking into consideration that their level of effort did not reach five or six at any time during the matches. Differently, in the DS group, some participants exhibited a level of effort compatible with this classification; thus, the practice could be considered as moderate-intensity PA. We speculate that the TD participants did not reach sufficient levels of RPE because the task was too easy for them, and they needed to be more challenged by the physical demands of the virtual game. 

The existing evidence on physical fitness in people with DS corroborates these findings. Ruiz-González et al., 2019 [26], in a systematic review, showed the potential benefit of physical interventions, specifically in strength and balance, in people with DS. However, the authors did not find studies with benefits in aerobic training interventions (i.e., the studies analyzed did not provide changes in the perception of effort and intensity of the exercise in the participants with DS), and the authors justify this unfavorable effects because of the monotony and lack of motivation with activities such as walking or jogging. Moreover, the same systematic review presented that the use of technology with nonimmersive virtual reality tasks could be useful for individuals with DS, and this could justify our positive results.

Functional mobility, flexibility, agility, strength, and performance may be improved by exergames in DS individuals, with the additional benefit of increased satisfaction and motivation, providing fun and engaging practice [27]. Endurance and physical fitness are other aspects shown to be improved using VR interventions with exergames [19]. It is important to point out that this population is exposed to risk factors for many comorbidities during their lifespan, considering the known pathophysiological alterations present in DS systems of the body and that the regular practice of PA contributes to a healthier life [28,29].

An interesting aspect of our sample of participants with DS is that they did not show a pattern of predominantly sedentary behavior, evidenced by the IPAQ results (20.6% of DS individuals were sedentary). Despite reporting more active habits than usually found in the DS population, the participants still presented less tolerance to the exercise provided by the game, evidencing the need for appealing alternatives to improve adequate engagement in PA from a young age to optimize physical fitness across the lifespan and prevent sedentary behavior from early ages. 

### 4.2. Second Phase: Motor Performance during Game Practice

The individuals in the DS group started the protocol with worse performance than the TD group, with greater numbers of errors and inconsistencies, until a later stage, when they improved and approached the values shown by the TD group (on the 4th day of the protocol). 

The movement pattern of DS individuals is reported in the available literature as being typically slower than in TD individuals (i.e., they take more time to initiate movements and to complete the tasks required, presenting slower movement execution) [30,31]. Despite this, DS individuals can improve their movements and performance with practice, even when the task apparently has little room for improvement [31,32]. Our results show that even with worse performance at the beginning of the protocol, after familiarization with the game task and over the days of practice (in the case of our study, on the fourth day), the DS participants were able to improve in the virtual task and reach the TD group’s level of performance. Menezes et al. [27], who analyzed individuals with DS using a VR maze task, emphasized that people with DS improved their performance with practice but underperformed in comparison with people with typical development, and further, they demonstrated difficulty in keeping the movement time with increasing task difficulty. 

Although this improvement with practice is a good outcome, an important aspect of learning a task is not only to facilitate performance in temporary effects (i.e., during acquisition, when the person is practicing to enhance their performance, reducing errors) but also to provide durable enhanced performance over a follow-up period (i.e., retention of the task learned). We conducted a follow-up period of 15 days after finishing the practice (10 days of acquisition) and found that DS participants maintained performance on the follow-up day, with no significant difference between the 10th day (i.e., final day of acquisition) with the 11th day (i.e., follow-up day after the 15 day break). Thus, the DS individuals maintained the ability to perform the task after a certain time without practicing. Additionally, after the fourth day of the protocol (i.e., when the participants reached the TD performance), they maintained good outcomes on all remaining days of the protocol, with no significant improvement but also with no deterioration in their performance. Kokol et al. [33], in a systematic review of serious game-based interventions for children with developmental disabilities, support our results. The authors presented studies with improvement of performance in highly practiced motor skills using Wii games in individuals with DS.

These results contribute information on the potential for motor improvement in people with DS, as they present the muscular and neural machinery necessary to perform movements with similar characteristics to those seen in individuals without DS. Thus, it is necessary to provide proper opportunities, with enough time and stimuli in order to optimize improvement in this population, taking into account the physiological alterations that lead to the movements and learning being slower in DS individuals than their TD peers [33,34].

### 4.3. Absolute and Variable Errors: Accuracy and Precision When Practicing a Timing Coincident Task

We analyzed two parameters of motor performance considering the MoveHero task: the absolute error (AE)—which is the representation of the movement accuracy—and the variable error (VE)—which is the representation of the precision of movements. The AE is the degree of proximity to the target and, thus, the value obtained is a reference related to the individual reaching the target or not. Differently, the VE identifies the precision of the movements, expressing the degree of consistency of the responses on the target related to the variability of the results [14]. 

We identified differences considering the accuracy and precision of the improvement in the DS individuals. Our results show that the participants presented more difficulty in precision than accuracy. Over the period of the protocol, the DS individuals improved in AE (i.e., improved their accuracy), reaching similar values as the TD group; however, for VE (i.e., precision), the DS group did not improve as much, and their performance continued to be worse than the TD group. The enhancement in performance considering their improvement in accuracy is related to their ability, with practice, to learn the task proposed, but the results regarding the precision showed that despite learning the task, the DS individuals present difficulties with adjustments to finer and more specific movements. 

Considering that lower values of precision (evidenced by the VE) represent less variability in movement, the DS individuals showed very similar patterns of errors during the task. This indicates that their adjustments and corrections of movements based on their misses (visualized by the game’s negative feedback to improve in the next attempt) were not successful. These results are in agreement with Monteiro et al. [14], who found that DS individuals experience difficulty practicing a simple timing coincident task. The authors presented and justified that this difficulty was due to allocating less time in the deceleration of reaching, having less anticipatory capacity, and having greater difficulty in executing the strategy used to accomplish the task.

Over the days of the second phase of the protocol, the DS group presented increases in VE when comparing the first day (D1) with the other 10 days (D2 to D11), which may imply that they came to understand the task better; that is, they varied their movements more to try to hit the spheres at the right time (i.e., when they reached the target). However, despite this improvement, they did not achieve the same pattern of precision as the TD individuals. 

We can speculate that this outcome is related to impairments in the anticipatory adjustments, arising from decreased motor proficiency because of the slower adaptation to environmental changes and specific motor task demands that DS individuals present [35]. In other words, although their performance improved with practice in the virtual task, their adaptations to the demand of the task were not able to reach the pattern of the TD individuals (i.e., considering the lower variability shown in the VE). However, considering the good outcomes of the DS individuals in AE, it was shown that these individuals can learn virtual motor tasks with practice, and enhance their performance similarly to TD individuals. The physical and cognitive particularities of DS individuals must be taken into consideration, and adequate opportunities should be provided, with different stimuli and longer periods of time, to enable them to learn the task and improve their motor efficiency. 

## 5. Limitations and Future Studies

Although we found interesting results, we can point out some limitations of the present study: (1) we performed the second phase of the protocol only with the DS individuals, limiting the possibility of comparisons between groups; (2) we did not perform quality of life and motivation assessments, which may be improved by the VR intervention, and studies addressing these components could be interesting to reinforce the benefits of VR for DS individuals; (3) we did not standardize the HR measurement, the instrument used varied according to family availability (smart watch, pulse oximeter, or cellphone application).

## 6. Conclusions

VR exergames are a great possibility to promote an alternative modality of PA and, consequently, a way out of the sedentary lifestyle of DS individuals, considering the results regarding the enhancement in the HR and RPE of the participants. Additionally, our results showed positive outcomes regarding motor performance, with significant improvement in the proposed virtual task with practice, showing that individuals with DS are able to improve their motor proficiency in the virtual environment with adequate stimuli.

The results of our study are relevant to the treatment of individuals with DS and add possibilities for therapies. We believe that exergames may positively influence rehabilitation programs for DS individuals, considering that they are a low-cost intervention that takes place at home, eliminating difficulties with traveling. Additionally, VR exergames cover a wide age group, allowing their use not only by children but also by adolescents and adults, encouraging these individuals to remain active with an activity that gives them pleasure and promotes well-being. 

Thus, we believe that the results of this study provide scientific support for the use of VR games at home by individuals with DS to reduce sedentary habits, improve fitness, and maintain the functionality of these individuals.

## Figures and Tables

**Table 1:** Characteristics of the Down syndrome (DS) and typical development (TD) groups.

	DS	TD
Participants	34	34
Mean age	18.9 (±6.1)	19.0 (±1.6)
Active ^1^	13 (38.2%)	10 (28.6%)
Very active ^1^	6 (17.6%)	5 (14.3%)
Irregularly active ^1^	8 (23.5%)	9 (25.7%)
Sedentary ^1^	7 (20.6%)	10 (28.6%)
Cellphone ^2^	32 (94.1%)	34 (100.0%)
Tablet ^2^	24 (70.6%)	18 (51.4%)
Computer/laptop ^2^	23 (67.6%)	32 (91.4%)
TV ^2^	33 (97.1%)	33 (94.3%)
Video game ^2^	4 (11.8%)	22 (62.9%)
<3 h/week ^3^	1 (2.9%)	0 (0%)
3–6 h/week ^3^	1 (2.9%)	1 (2.9%)
7–10 h/week ^3^	3 (8.8%)	3 (8.6%)
>10 h/week ^3^	29 (85.3%)	30 (85.7%)

^1^ IPAQ—International Physical Activity Questionnaire classification. ^2^ ED—electronic devices. ^3^ ST—screen time.

## Data Availability

Some or all data and models that support the findings of this study are available from the corresponding author upon reasonable request.

## Supplementary Materials

The following supporting information can be downloaded at: https://www.mdpi.com/article/10.3390/healthcare11131894/s1, Table S1: Phase one of the protocol: comparison of HR, RPE and motor performance (AE and VE) for conditions (within-groups) and between-groups. Table S2: Phase two of the protocol: comparison of HR, RPE and motor performance (AE and VE) for conditions (within-group).
