# Supplementary material for: Analysis of Heart Rate, Perception of Physical Effort and Performance of Individuals with Down Syndrome Submitted to a Protocol of Virtual Games for Home-Based Telerehabilitation
Source: Healthcare (Basel). 2023 Jun 30;11(13):1894. doi: 10.3390/healthcare11131894 (PMC10341266; doi:10.3390/healthcare11131894)
Supplement: Supplementary file 1 [file healthcare-11-01894-s001.zip › Supplementary_Material - SD longitudinal.docx]

Supplementary Material

Table S2. Phase two of the protocol: comparison of HR, RPE and motor performance (AE and VE) for conditions (within-group).

| Variables |  |  |  |  | | | | |  |  |
| --- | --- | --- | --- | --- | --- | --- | --- | --- | --- | --- |
|  |  | Rest  Mean (SE) | Match1  Mean (SE) | p-value (Rest X  Match 1) | Match2  Mean (SE) | p-value (Match1 X Match2) | Match3  Mean (SE) | p-value  (Match2 X Match3) | p-value (Rest X Match 3) |  |
| HR | D1 | 74.52 (2.26) | 85.41 (2.26) | **p<0.05** | 85.79 (1.60) | 0.392 | 86.40 (2.26) | **0.007** | **p<0.05** |  |
|  | D5 | - | 83.24 (2.87) | - | 81.43 (2.87) | 0.656 | 87.14 (2.81) | 0.156 | 0.332 |  |
|  |  |  | 0.552 |  | 0.184 |  | 0.837 |  |  |  |
|  | D10 | - | 82.81 (3.29) | - | 86.00 (3.29) | 0.494 | 88.63 (3.29) | 0.573 | 0.212 | p-value  (D1 x D10) |
|  |  |  | 0.922 |  | 0.296 |  | 0.731 |  |  | 0.201 |
|  | D11 | - | 83.69 (3.29) | - | 87.76 (3.29) | 0.383 | 89.50 (3.29) | 0.707 | 0.212 | p-value  (D1xD11) |
|  |  |  | 0.851 |  | 0.707 |  | 0.851 |  |  | 0.070 |
| RPE | D1 | 0.09 (0.22) | 3.12 (0.22) | **p<0.05** | 3.32 (0.16) | 0.443 | 4.06 (0.22) | **0.006** | **p<0.05** |  |
|  | D5 | -  [672.1; 752.7] | 3.29 (0.28) | - | 0.00 (0.28) | **p<0.05** | 3.41 (0.28) | **p<0.05** | 0.752 |  |
|  |  |  | 0.636 |  | **p<0.05** |  | 0.063 |  |  |  |
|  | D10 | - | 2.89 (0.32) | - | 3.06 (0.32) | 0.678 | 3.63 (0.32) | 0.213 | 0.097 | p-value  (D1 x D10) |
|  |  |  | 0.333 |  | **p<0.05** |  | 0.607 |  |  | **0.011** |
|  | D11 | - | 2.76 (0.32) | - | 3.13 (0.32) | 0.407 | 3.69 (0.32) | 0.213 | **0.038** | p-value  (D1xD11) |
|  |  |  | 0.782 |  | 0.890 |  | 0.890 |  |  | **0.011** |
|  |  | Match 0  Mean (SE) | Match 1  Mean (SE) | p-value  (Match0 X Match 1) | Match 2  Mean (SE) | p-value  (Match1 X Match2) | Match 3  Mean (SE) | p-value  (Match2 X Match3) | p-value (Match0 X Match 3) |  |
| AE | D1 | 2894.06 (86.15) | 1193.36 (84.87) | **p<0.05** | 1072.69 (84.87) | 0.315 | 996.44 (84.87) | 0.525 | **p<0.05** |  |
|  | D5 | - | 670.90 (107.44) |  | 633.89 (107.99) | 0.809 | 612.23 (107.99) | 0.887 | 0.701 |  |
|  |  |  | **p<0.05** | - | **0.001** |  | **0.005** |  |  |  |
|  | D10 | - | 672.81 (137.23) |  | 579.19 (120.03) | 0.608 | 510.15 (120.03) | 0.658 | 0.373 | p-value  (D1xD10) |
|  |  |  | 0.991 | - | 0.735 |  | 0.528 |  |  | **p<0.05** |
|  | D11 | - | 749.65 (123.72) |  | 625.72 (123.22) | 0.479 | 619.03 (123.72) | 0.970 | 0.456 | p-value  (D1xD11) |
|  |  |  | 0.678 | - | 0.787 |  | 0.529 |  |  | **p<0.05** |
| VE | D1 | 363.89 (58.76) | 744.19 (57.89) | **p<0.05** | 754.61 (57.89) | 0.899 | 763.10 (57.89) | 0.917 | **p<0.05** |  |
|  | D5 | - | 729.78 (73.66) | - | 742.85 (73.66) | 0.900 | 799.52 (77.44) | 0.596 | 0.514 |  |
|  |  |  | 0.878 |  | 0.900 |  | 0.706 |  |  |  |
|  | D10 | - | 841.35 (81.87) | - | 754.51 (81.86) | 0.453 | 764.51 (81.86) | 0.931 | 0.507 | p-value  (D1xD10) |
|  |  |  | 0.311 |  | 0.916 |  | 0.756 |  |  | **0.019** |
|  | D11 |  | 764.43 (84.39) |  | 772.98 (84.39) | 0.943 | 781.98 (84.39) | 0.940 | 0.883 | p-value  (D1xD11) |
|  |  |  | 0.513 |  | 0.875 |  | 0.882 |  |  | **0.040** |
