# Supplementary material for: Analysis of Heart Rate, Perception of Physical Effort and Performance of Individuals with Down Syndrome Submitted to a Protocol of Virtual Games for Home-Based Telerehabilitation
Source: Healthcare (Basel). 2023 Jun 30;11(13):1894. doi: 10.3390/healthcare11131894 (PMC10341266; doi:10.3390/healthcare11131894)
Supplement: Supplementary file 1 [file healthcare-11-01894-s001.zip › Supplementary_Material - SDxTD.docx]

Supplementary Material

Table S1. Phase one of the protocol: comparison of HR, RPE and motor performance (AE and VE) for conditions (within-groups) and between-groups.

| Variables |  |  |  |  | | | | |  |
| --- | --- | --- | --- | --- | --- | --- | --- | --- | --- |
|  |  | Rest  Mean (SE) | Match1  Mean (SE) | p-value (Rest X  Match 1) | Match2  Mean (SE) | p-value (Match1 X Match2) | Match3  Mean (SE) | p-value  (Match2 X Match3) | p-value (Rest X Match 3) |
| HR | DS | 74.52 (2.26) | 85.41 (2.26) | **p<0.001** | 85.79 (1.60) | 0.392 | 86.40 (2.26) | **0.007** | **p<0.05** |
|  | TD | 76.50 (2.26) | 84.52 (2.03) | **0.008** | 85.32 (1.60) | 0.757 | 89.18 (2.26) | 0.164 | **p<0.05** |
|  |  | 0.537 | 0.770 |  | 0.835 |  | 0.384 |  |  |
| RPE | DS | 0.09 (0.22) | 3.12 (0.22) | **p<0.05** | 3.32 (0.16) | 0.443 | 4.06 (0.22) | **0.006** | **p<0.05** |
|  | TD | 0.52 (0.22) | 2.33 (0.20) | **p<0.05** | 2.82 (0.16) | 0.051 | 3.23 (0.22) | 0.125 | **p<0.05** |
|  |  | 0.172 | **0.008** |  | **0.023** |  | **0.008** |  |  |
|  |  | Match 0  Mean (SE) | Match 1  Mean (SE) | p-value  (Match0 X Match 1) | Match 2  Mean (SE) | p-value  (Match1 X Match2) | Match 3  Mean (SE) | p-value  (Match2 X Match3) | p-value (Match0 X Match 3) |
| AE | DS | 2894.06 (86.15) | 1193.36 (84.87) | **p<0.05** | 1072.69 (84.87) | 0.315 | 996.44 (84.87) | 0.525 | **p<0.05** |
|  | TD | 1208.12 (98.98) | 848.87 (86.15) | **0.006** | 811.00 (90.35) | 0.762 | 693.53 (90.35) | 0.358 | **p<0.05** |
|  |  | **p<0.05** | **0.005** |  | **0.035** |  | **0.015** |  |  |
| VE | DS | 363.90 (58.76) | 744.19 (57.89) | **p<0.05** | 754.61 (57.89) | 0.899 | 763.10 (57.89) | 0.917 | **p<0.05** |
|  | TD | 780.72 (67.51) | 750.04 (58.76) | 0.732 | 622.21 (61.63) | 0.134 | 592.45 (61.67) | 0.733 | **0.040** |
|  |  | **p<0.05** | 0.944 |  | 0.118 |  | **0.044** |  |  |
